# Cross-linked chitosan/lysozyme hydrogels with inherent antibacterial activity and tuneable drug release properties for cutaneous drug administration

**DOI:** 10.1080/14686996.2023.2167466

**Published:** 2023-02-21

**Authors:** Wing-Fu Lai, Obireddy Sreekanth Reddy, Dahong Zhang, Haicui Wu, Wing-Tak Wong

**Affiliations:** a Department of Urology, Zhejiang Provincial People’s Hospital, Affiliated People’s Hospital, Hangzhou Medical College, Zhejiang, China; b Department of Applied Biology and Chemical Technology, Hong Kong Polytechnic University, Hong Kong Special Administrative Region, China; c Department of Chemistry, Sri Krishnadevaraya University, Anantapur, India

**Keywords:** Chitosan, lysozyme, cutaneous administration, drug delivery, sustained release

## Abstract

Gels with high drug release sustainability and intrinsic antibacterial properties are of high practical potential for cutaneous drug administration, particularly for wound care and skin disease treatment. This study reports the generation and characterization of gels formed by 1,5-pentanedial-mediated crosslinking between chitosan and lysozyme for cutaneous drug delivery. Structures of the gels are characterized by using scanning electron microscopy, X-ray diffractometry and Fourier-transform infrared spectroscopy. An increase in the mass percentage of lysozyme leads to an increase in the swelling ratio and erosion susceptibility of the resulting gels. The drug delivery performance of the gels can be changed simply by manipulating the chitosan/lysozyme mass-to-mass ratio, with an increase in the mass percentage of lysozyme leading to a decline in the encapsulation efficiency and drug release sustainability of the gels. Not only do all gels tested in this study show negligible toxicity in NIH/3T3 fibroblasts, they also demonstrate intrinsic antibacterial effects against both Gram-negative and Gram-positive bacteria, with the magnitude of the effect being positively related to the mass percentage of lysozyme. All these warrant the gels to be further developed as intrinsically antibacterial carriers for cutaneous drug administration.

## GRAPHICAL ABSTRACT

**Figure:**
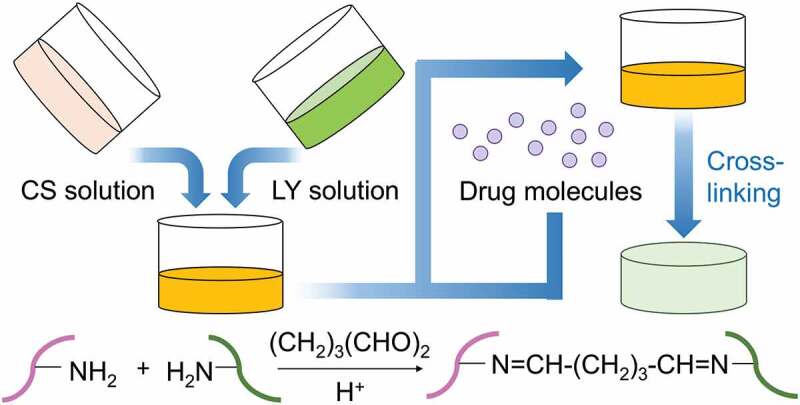

## 1. Introduction

Gels have been playing an important role in cutaneous drug administration because they can form close contact with mucosal surface and enable sustained release of the loaded drugs into the application site [1–6]. Till now gels in different forms, ranging from nanoparticles to fibres, have been generated [7–12]. For example, an earlier study has reported an ionically crosslinked hydrogel formed by using carmellose sodium and chitosan (CS) for delivery of minocycline hydrochloride (MH) in wound treatment [13]. The gel has been found to enhance the rate of wound closure and to protect the wound in mice from infection. Upon covalent crosslinking of CS with polyethylenimine and ionic crosslinking with carmellose sodium, a bioinspired, sustained-release material in response to internal signals for biphasic chemical sensing in wound therapy has been generated [14]. In preclinical trials, not only has the gel increased the rate of wound closure, but it has also been shown to reduce inflammatory cell infiltration and to enhance collagen deposition [14]. Importantly, its biphasic chemical sensing capacity has been demonstrated to potentially prevent possible occurrence of skin hyperpigmentation caused by MH in wound therapy [14]. Along with its ease of fabrication, high biocompatibility and high drug release sustainability [14], the gel has exhibited high potential to be further exploited as an intelligent solid-state device for wound care.

In fact, as far as wound treatment is concerned, infection is one of the major areas of concern. Prevention from infection has been found to successfully facilitate wound healing [15,16]. Over the years, different wound dressings displaying anti-infective properties have been generated [17–21]; however, many of them rely on the addition of exogenous agents into the dressings to act against infection. The release of these agents from the gels into the skin site may interfere with the action of other therapeutic agents used in treatment and may cause adverse reactions [22,23]. This problem is compounded by the situation that the drug release sustainability of the gels is generally poor [24–26]. This causes rapid release of the loaded anti-infective agents from the gels and hence loss of anti-infective activity shortly after application to the skin. To address these problems, development of sustained-release gels that show intrinsic anti-infective properties is of practical importance. In this study, we develop an intrinsically antibacterial gel from hen egg white lysozyme (LY) and CS for cutaneous drug administration. The gel is strengthened by using covalent crosslinking, which enhances the capacity of the gel to combat swelling and erosion and hence show enhanced drug release sustainability for treatment of skin disorders.

## 2. Materials and methods

### 2.1 Materials

LY, and various other chemicals were purchased from Macklin Chemical Co., Ltd. (Shanghai, China). Dulbecco’s Modified Eagle’s Medium (DMEM; Gibco, Grand Island, U.S.A), penicillin G-streptomycin sulphate (Life Technologies Inc., U.S.A), and foetal bovine serum (FBS; Hangzhou Sijiqing Biological Engineering Materials Co., Ltd., China) were used for cell culture. Trypsin-ethylenediaminetetraacetic acid (0.25% trypsin-EDTA) was purchased from Invitrogen (Carlsbad, California, U.S.A).

### 2.2 Gel preparation

CS was dissolved in a 5% (w/v) acetic acid solution. 10 mL of the solution was mixed with an equal volume of a 5% (w/v) aqueous solution of LY. 2 mL of 1,5-pentanedial was added. Gelation of the solution mixture was performed at ambient conditions. The resulting gel (namely CY gel) was designated as CY11. Gels having other mass-to-mass ratios of CS and LY were generated, with the volumes of different reagents being shown in Table 1.

**Table 1.** Volumes of different reagents used for gel preparation.

Gel	CS:LY ratio	CS solution (mL)	LY solution (mL)	1,5-pentanedial (mL)
CY10	1:0	20	0	2
CY31	3:1	15	5	2
CY11	1:1	10	10	2
CY13	1:3	5	15	2

### 2.3 Fourier-transform infrared (FTIR) spectroscopy

The structures of CS, LY and CY gels were determined at ambient conditions by using an FTIR spectrometer (Spectrum 2000; PerkinElmer, Norwalk, Connecticut, U.S.A). The potassium bromide disk technique was applied for sample preparation. Spectra were collected at a resolution of 2 cm^−1^, and reported as an average of 16 scans.

### 2.4 Scanning electron microscopy (SEM) analysis

Gels were lyophilized and sputter-coated with gold for SEM analysis. A scanning electron microscope (JSM-6380; JEOL, Tokyo, Japan) operated at an accelerating voltage of 10 kV was applied to image the surface morphology of the gels.

### 2.5 X-ray diffractometry (XRD) analysis

Diffraction patterns of the gels were obtained in a range of 5-80° by using a D8 Advance diffractometer (Bruker-AXS; Bruker, Karlsruhe, Germany) with Cu-Kα radiation (λ = 1.5406 Å). All diffraction patterns were collected at 40 kV and 40 mA.

### 2.6 Evaluation of the swelling capacity and erosion susceptibility

The swelling properties and erosion profiles of the gels were determined using the procedures as previously reported [13]. The swelling ratio was calculated using the following equation:

(1) $$Swelling\;\;ratio=\frac{m_{s}}{m_{d}}$$

where *m*_*s*_ and *m*_*d*_ represent the mass of the swollen gel and the mass of the dried gel, respectively.

### 2.7 Evaluation of acute and chronic toxicity in vitro

NIH/3T3 fibroblasts were cultured as previously delineated [27]. The CellTiter 96 Aqueous Non-radioactive Cell Proliferation Assay (MTS assay; Promega Corp., Madison, Wisconsin, U.S.A) was performed to evaluate the *in vitro* toxicity of CS, LY and the lyophilized CY gels as reported in an earlier study [28].

### 2.8 Evaluation of antibacterial properties of CY gels

*Staphylococcus aureus* and *Escherichia coli* were cultured in the Luria-Bertani (LB) broth for 18 h. Their concentration was adjusted to 1 × 10^8^ CFU/mL, and then diluted into 1 × 10^6^ CFU/mL. Agar was added to another LB broth until the concentration of 0.8% (w/v) was reached to obtain soft agar. After autoclave, 18 mL of soft agar was mixed with 2 mL of the bacteria-containing LB broth. A lyophilized gel was put in the centre of the agar plate. After incubation at 37°C overnight, the area of the clear zone was measured.

### 2.9 Determination of transepidermal water loss (TEWL) after skin treatment with CY gels

Porcine ears were purchased from a local slaughterhouse. Hairs from the skin were removed by using a clipper. Blunt dissection was conducted to excise the full-thickness dorsal skin. Upon 5-h treatment of the skin with the gel, a Tewameter® 300 evaporimeter probe (Courage and Khazaka, Germany) was put on the skin surface to measure the TEWL values.

### 2.10 Determination of the encapsulation efficiency (EE)

10 mg of MH was added to 20 mL of a CS/LY solution mixture prior to the addition of 2 mL of 1,5-pentanedial as described above. CY10, CY31, CY11 and CY13 were designed as CY10-MH, CY31-MH, CY11-MH and CY13-MH, respectively, after the drug loading process. The concentration of the unloaded drug was measured by using an ultraviolet-visible (UV-Vis) spectrophotometer at λ_max_ of 280 nm. The EE was estimated using the following equation:

(2) $$EE\left(\%\right)=\frac{m_{l}}{m_{t}}\times 100\%$$

where *m*_*l*_ is the mass of MH encapsulated successfully by the gel, and *m*_*t*_ is the total mass of MH added during the drug encapsulation process.

### 2.11 Determination of the sustainability and kinetics of drug release

Evaluation of the drug release sustainability of the gels was performed based on a previously reported protocol [29]. In brief, 13 g of a MH-loaded gel was placed in 30 mL of phosphate-buffered saline (PBS) (pH 7.4), and was incubated at 25°C. At regular time intervals, 1 mL of the release medium was withdrawn, and replenished by the same volume of PBS. The amount of MH released from the gel was measured by using a UV-Vis spectrophotometer at λ_max_ of 280 nm. The percentage of cumulative drug release was calculated by using the following equation:

(3) $$Cumulative\;release\;\left(\%\right)=\;\frac{\sum_{t=0}^{t}m_{t}}{m_{\infty }}\times 100\%$$

where *m*_t_ is the mass of MH released from the gel at time *t*, and *m*_*∞*_ is the total mass of MH loaded into the gel. The release curve was fitted into different kinetic models (including the zero-order model, the first-order model, the Higuchi model and the Korsmeyer-Peppas model) to analyse the mechanism of drug release.

## 3. Results and discussion

### 3.1 Fabrication of CY gels and structural characterization

**Figure 1.**
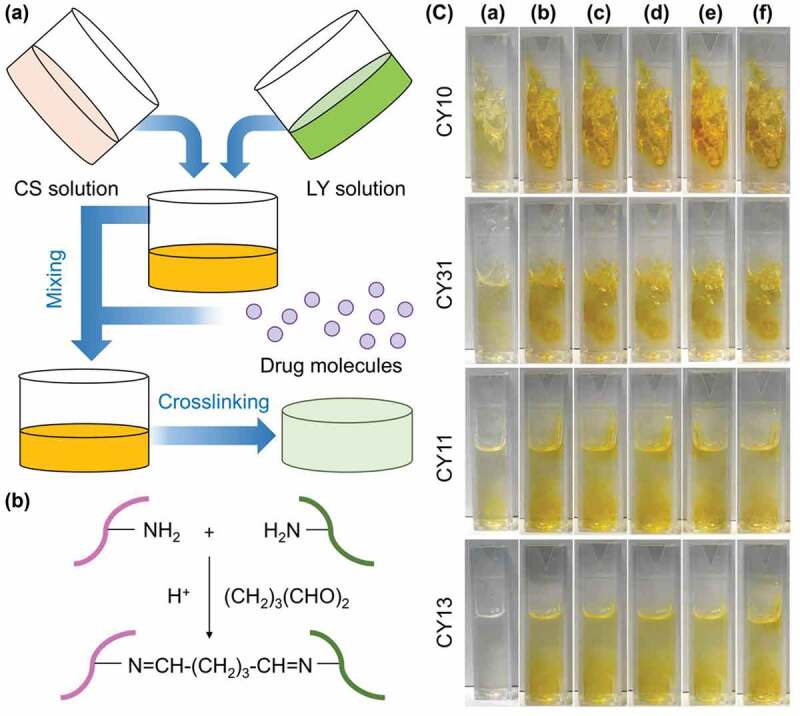
(a) A schematic diagram showing the procedures for the fabrication of a CY gel. (b) Schematic representation of the 1,5-pentanedial-mediated covalent crosslinking reaction held between CS and LY. The pink molecule and green molecule represent a CS molecule and an LY molecule, respectively. (c) Photos depicting changes in the reaction mixture during the gelation process exhibited by different CY gels. The photos are taken at (a) 0 min, (b) 40 min, (c) 80 min, (d) 120 min, (e) 160 min, and (f) 200 min after the addition of 1,5-pentanedial.

1,5-pentanedial is adopted to mediate the crosslinking reaction between the free amino groups of CS (and LY) and the aldehyde groups of 1,5-pentanedial to generate a gel for cutaneous drug administration (Figure 1) [30–35]. CS has low toxicity, high biocompatibility, and high biodegradability [36,37]. It has been widely adopted for preparation of hydrogels for drug delivery purposes [38–42]. On the other hand, LY shows antibacterial properties, and is particularly effective in working against Gram-positive bacteria by breaking down the peptidoglycan layers of the bacterial membrane [43]. Along with the intrinsic antibacterial properties of CS [44–48], the gels generated by using CS and LY are expected to not only enable sustained drug release but also show antibacterial properties.

**Figure 2.**
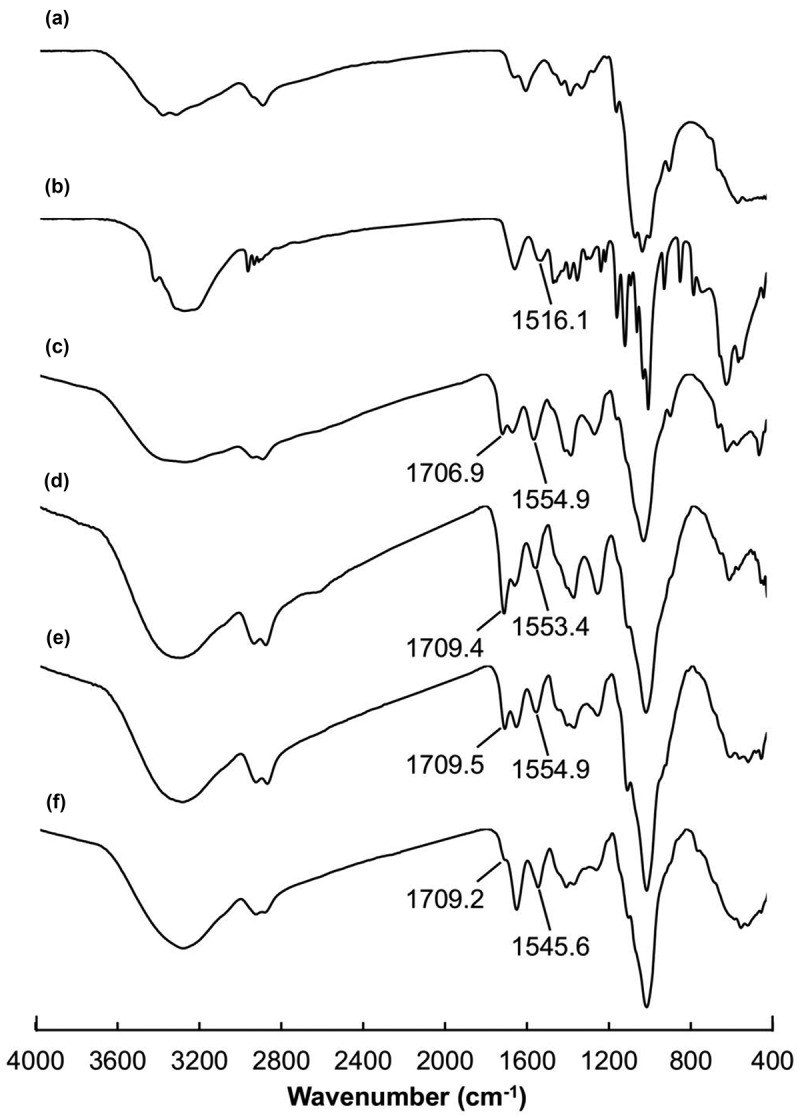
FTIR spectra of (a) CS, (b) LY, (c) CY10, (d) CY31, (e) CY11, and (f) CY13.

**Figure 3.**
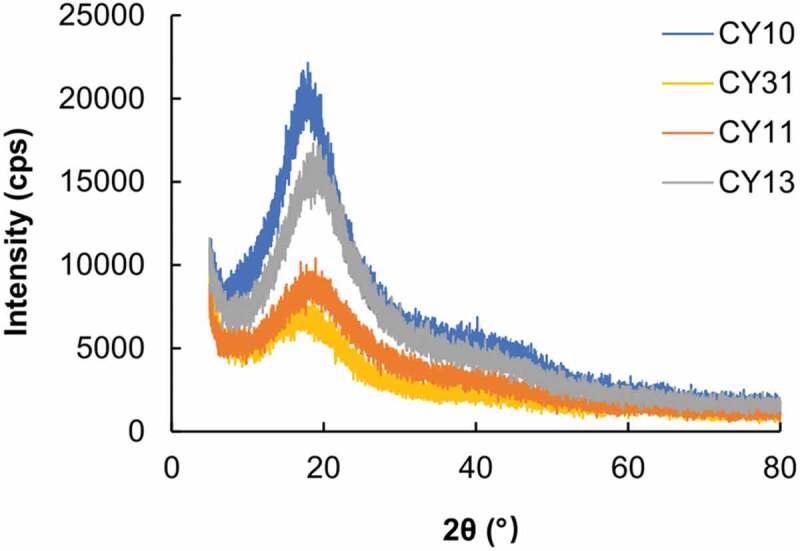
XRD curves of the CY gels.

The structures of CS, LY and the gels formed are characterized by using FTIR spectroscopy (Figure 2). In the FTIR spectrum of LY, the amide band at 1516 cm^−1^ is closely related to the secondary structure of LY [49,50]. A slight shift in the position of this band is observed after 1,5-pentanedial-mediated covalent crosslinking between LY and CS. This suggests that the process of crosslinking causes a change in the secondary structure of the protein. In addition, upon 1,5-pentanedial-mediated covalent crosslinking, a peak at 1707 cm^−1^ is found. This peak is absent in the spectrum of CS, and is attributed to the stretching vibration of the imine bond formed between the amino group of CS and the aldehyde group of 1,5-pentanedial. The structures of CY gels are further characterized by XRD (Figure 3). CS is expected to have a semi-crystalline structure which leads to the presence of a crystalline peak at 2θ = 20° and an extensive peak at 2θ in the range of 30-50°. This is consistent with what reported in an earlier study [51]. The extensive peak is attributed to the amorphous phase of CS. These peaks are present in the XRD patterns of CY gels, indicating that the semi-crystalline structure of CS is retained upon the crosslinking process.

### 3.2 Toxicity and antibacterial properties of CY gels

**Figure 4.**
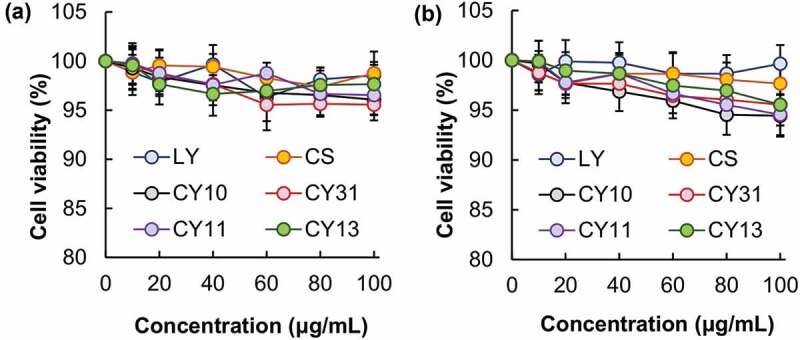
Viability of NIH/3T3 fibroblasts after 5-h treatment with CS, LY and different CY gels, (a) before and (b) after 24 h post-treatment incubation.

**Figure 5.**
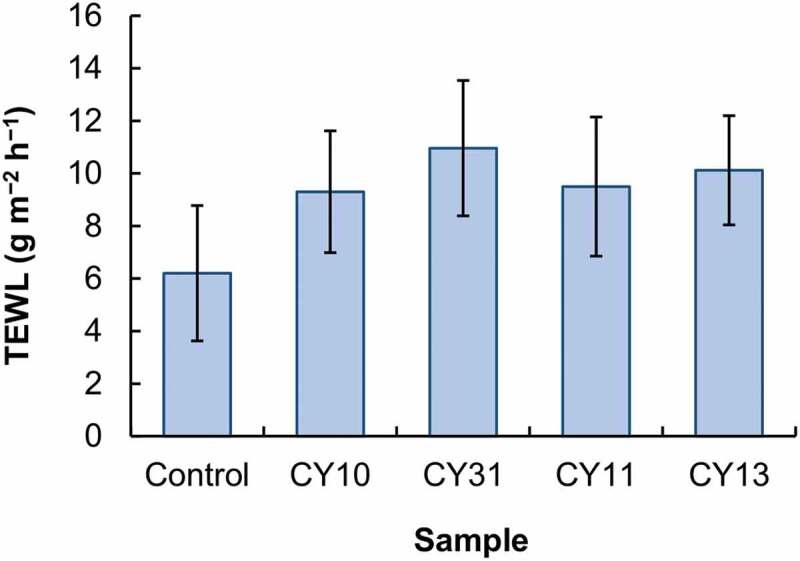
The TEWL values of the skin after 5-h treatment with different CY gels. No treatment is applied to the control group.

The toxicity of CS and LY is studied in NIH/3T3 fibroblasts (Figure 4). After 5-h treatment with the culture medium containing either CS or LY at different concentrations, no apparent loss of cell viability is observed. This confirms the absence of acute toxicity of both CS and LY *in vitro*. Formation of gels from CS and LY leads to no observable increase in acute cytotoxicity. To determine the chronic toxic effect possibly brought about by CS, LY or the gels formed in NIH/3T3 cells, the viability of the treated cells is determined 24 h after treatment. No apparent increase in the loss of cell viability is found in all concentrations tested. This suggests that CS, LY, and the gels formed show high safety profiles for use in drug delivery. Apart from the absence of toxic effects, gels used for cutaneous drug administration should show minimal influence on the integrity of the skin. This is examined by using TEWL as an indicator of skin integrity (Figure 5). TEWL is adopted because its change can be caused by alterations in multiple features (including the thickness of stratum corneum, the skin lipid content, and the number of lamellar bodies) of the skin [52]. After treatment of the skin with the CY gels, no significant change in the value of TEWL is observed. This suggests that the influence of the gels, regardless of the mass percentage of CS and LY, on the integrity of the skin is negligible.

**Figure 6.**
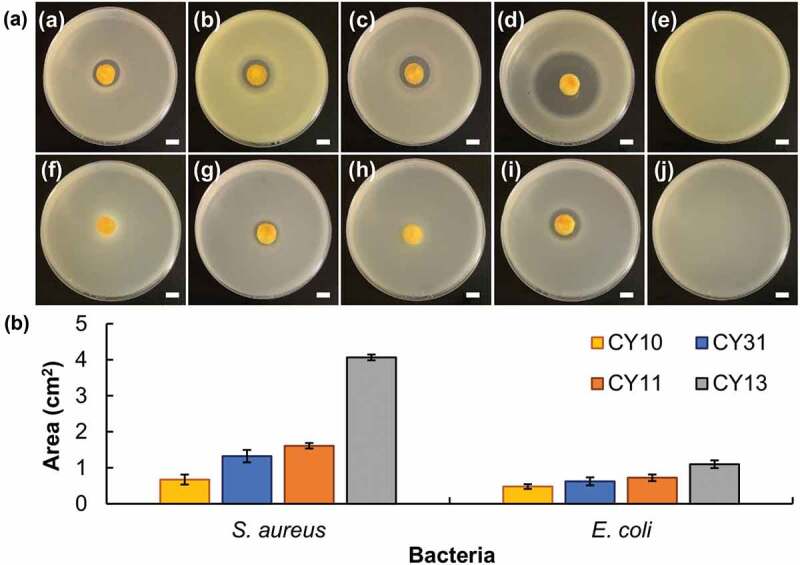
(a) Images showing the zone of inhibition induced by (a, f) CY10, (b, g) CY31, (c, h) CY11, and (d, i) CY13 for (a – e) *S. aureus* and (f – j) *E. coli*. (e) and (j) are the controls in which no treatment is administered. Scale bar = 1 cm. (b) Area percentages of the zone of inhibition induced by different CY gels. Data are presented as the means ± SD of triplicate experiments.

The intrinsic antibacterial properties of CY gels are examined by using *Staphylococcus aureus* (Gram-positive bacteria) and *Escherichia coli* (Gram-negative bacteria) as models (Figure 6). A decrease in the CS/LY mass-to-mass ratio of the CY gel leads to an increase in the antibacterial capacity. Compared to that on *Escherichia coli*, the growth-inhibiting activity of the CY gels on *Staphylococcus aureus* is much more significant. This may be explained by the fact that LY is particularly effective in inhibiting Gram-positive bacteria by causing the breakdown of the peptidoglycan layers of the bacterial membrane [43]. Such anti-bacterial properties render CY gels particularly favorable for use in cutaneous drug administration to address skin problems (e.g., wound closure) in which infection is one factor to be tackled.

### 3.3 Performance as carriers for cutaneous drug administration

**Figure 7.**
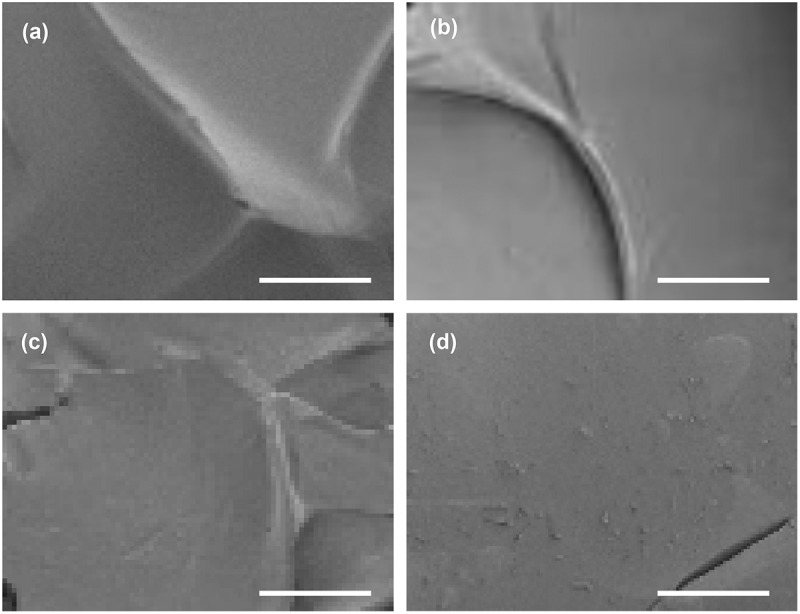
SEM images of the surface morphology of (a) CY10, (b) CY31, (c) CY11, and (d) CY13. Scale bar = 100 μm.

**Figure 8.**
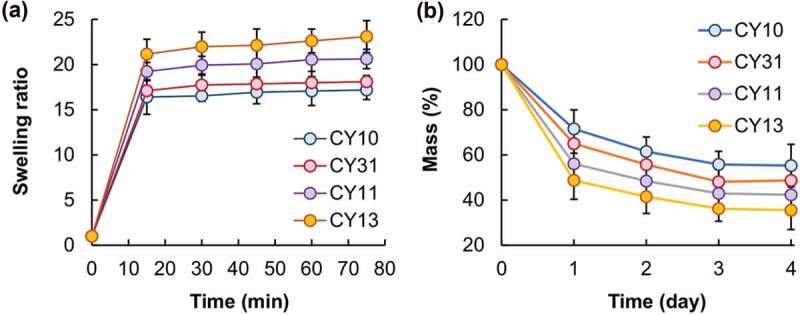
Plots showing the (a) swelling capacity and (b) erosion susceptibility of the CY gels.

**Figure 9.**
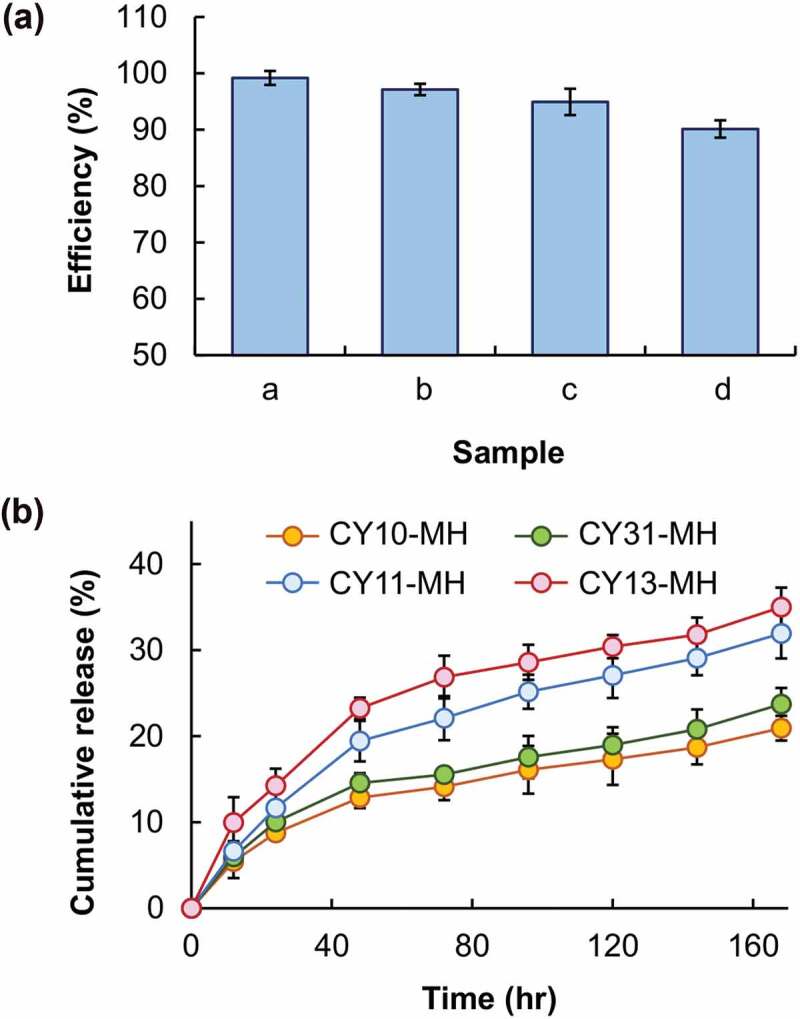
(a) The EE of different MH-loaded CY gels: (a) CY10-MH, (b) CY31-MH, (c) CY11-MH, and (d) CY13-MH. (b) Profiles of drug release from different MH-loaded CY gels.

The morphological features of the CY gels are examined by using SEM (Figure 7). All gels possess a smooth surface. Although changes in the CS/LY mass-to-mass ratio have no significant influence on the surface morphology of the gels, an increase in the mass percentage of LY leads to an increase in the swelling capacity of the gel. This may be because, compared to CS, LY is more hydrophilic in nature. This leads to an increase in the hydrophilicity of the generated gel when the mass percentage of LY increases, thereby causing an increase in the swelling capacity and erosion susceptibility (Figure 8). This explains the observation that a decrease in the CS/LY mass-to-mass ratio results in an increase in the rate of drug release, even though changing the mass percentage of CS and LY appears to have no apparent effect on the EE, which is approximated to be above 90% for all gels tested (Figure 9).

The curves of drug release are fitted into various kinetic models (including the zero-order model, the first-order model, the Higuchi model and the Korsmeyer-Peppas model). Based on the calculated regression coefficient (*r*^*2*^) values (Table 2), the release profiles of the tested gels fit the Higuchi model the most, regardless of the mass percentage of CS and LY. Drug release from all MH-loaded CY gels, therefore, involves the penetration of the release medium into the gel matrix. The mass-to-mass ratio of CS and LY has no significant effect on the mechanism of drug release. The release exponents (*n*), as calculated by using the Korsmeyer-Peppas equation, are 0.446, 0.446, 0.496 and 0.418 for CY10-MH, CY31-MH, CY11-MH and CY13-MH, respectively. Based on the *n* values, solvent diffusion is much more significant than polymer chain relaxation during the drug release process. The kinetics of drug release in all of the tested gels, regardless of the mass percentage of CS and LY, is thus controlled mainly by diffusivity [53].

**Table 2.** Release kinetic parameters of different MH-loaded CY gels.

Gel	Korsmeyer-Peppas		Zero-order		First-order		Higuchi	
	*n*	*r*^*2*^	*K*_*0*_	*r*^*2*^	*K*_*1*_	*r*^*2*^	*K*_*H*_	*r*^*2*^
CY10-MH	0.446	0.993	0.145	0.699	0.002	0.753	1.627	0.988
CY31-MH	0.446	0.989	0.162	0.699	0.002	0.757	1.812	0.984
CY11-MH	0.496	0.988	0.224	0.767	0.003	0.843	2.497	0.988
CY13-MH	0.418	0.984	0.252	0.626	0.003	0.743	2.838	0.973

## 4. Conclusions

Sustained-release gels having intrinsic antibacterial properties are of practical importance for cutaneous drug administration. In this study, we report a covalently crosslinked gel generated from LY and CS for drug encapsulation and sustained drug release. The swelling capacity and erosion susceptibility of the gels can be easily changed by manipulating the CS/LY mass-to-mass ratio. While the gels show negligible toxicity *in vitro* and have little effect on the integrity of the skin, they effectively inhibit the growth of bacteria. The antibacterial effect is particularly significant in Gram-positive bacteria. Based on the results presented in this study, CY gels show high potential to be developed and optimized as antibacterial sustained-release carriers with tuneable drug delivery performance for drug administration to treat diverse skin disorders in the future.
